# Food-Consumption Profiles and Context-Specific Associations Among University Students: An Exploratory Multicountry Person-Centred Clustering and CHAID Analysis

**DOI:** 10.3390/nu18172869

**Published:** 2026-09-02

**Authors:** Luis Moral-Moreno, Ivonne Vizcarra-Bordi, Alejandra Donají Benítez-Arciniega, Carmen Liliana Ceballos-Juárez, Edna Graciela García-Aguirre, Hilda Carmen Vargas-Cancino, David Eduardo Velázquez-Muñoz, Lucía Matilde Collado-Medina, Sergio Moctezuma-Pérez, Humberto Thomé-Ortiz

**Affiliations:** 1 Departamento de Didáctica de la Expresión Musical, Plástica y Corporal, Centro de Enseñanza Superior Don Bosco, María Auxiliadora 9, 28040 Madrid, Spain; 2 Instituto de Ciencias Agropecuarias y Rurales (ICAR), Campus El Cerrillo Piedras Blancas, Universidad Autónoma del Estado de México, Toluca 50295, Estado de México, Mexico; smoctezumap@uaemex.mx (S.M.-P.); hthomeo@uaemex.mx (H.T.-O.); 3 Facultad de Medicina, Universidad Autónoma del Estado de México, Avenida Paseo Tollocan, Esquina Jesús Carranza S/N, Colonia Universidad, Toluca 50180, Estado de México, Mexico; abeniteza@uaemex.mx (A.D.B.-A.); cceballosj001@alumno.uaemex.mx (C.L.C.-J.); egarciaa023@alumno.uaemex.mx (E.G.G.-A.); 4 Instituto de Estudios Sobre la Universidad (IESU), Universidad Autónoma del Estado de México, Avenida Paseo Tollocan Poniente 1402, Puertas F1 y F2, Colonia Universidad, Toluca 50110, Estado de Mexico, Mexico; hcvargasc@uaemex.mx (H.C.V.-C.); lmcolladom@uaemex.mx (L.M.C.-M.); 5 Facultad de Odontología, Universidad Autónoma del Estado de México, Avenida Paseo Tollocan, Esquina Jesús Carranza S/N, Colonia Universidad, Toluca 50130, Estado de México, Mexico; develazquezm@uaemex.mx

**Keywords:** IASE, dietary heterogeneity, dietary behaviour, health promotion, physical activity, cross-cultural comparison, higher education, healthy eating

## Abstract

**Background:** University students often report suboptimal dietary practices, but overall diet-quality scores may conceal differences in food-group consumption. This study explored whether distinguishable food-consumption profiles could be identified among university students from Chile, Mexico, Spain, and Italy, and examined, exploratorily, the characteristics associated with profile membership. **Methods:** In this cross-sectional study, 686 university students completed a dietary assessment using nine component scores of the Healthy Eating Index adapted for the Spanish population (IASE). Profiles were derived by Ward’s hierarchical clustering with k-means refinement and assessed for split-half stability; Exhaustive CHAID explored characteristics associated with profile membership. **Results:** Internal validity indices favoured no candidate solution (silhouette 0.113–0.126 across two- to five-cluster solutions), and split-half stability was limited throughout, including for the retained four-cluster solution (mean Adjusted Rand Index = 0.30). Four profiles were nonetheless retained: Healthy and Prudent (24.8%), Low Plant-Food (13.0%), Mixed Dietary (36.6%), and Protein-Oriented (25.7%); all occurred in every country. Country was the first CHAID split, followed by living arrangement (Chile/Mexico), year of study (Spain), and post-pandemic PA change (Italy). The model did not outperform the majority-class baseline; the segmentation is therefore exploratory, not predictive. **Conclusions:** A four-cluster configuration could be derived in each setting, but validity and stability indices indicate it is not robust; country/academic-programme—confounded with institution—was the only characteristic with a non-negligible, though modest, association with profile membership. Findings are hypothesis-generating and require replication in larger, representative, longitudinal samples before profiles are treated as an established typology.

## 1. Introduction

The transition to university represents a critical developmental period in which greater autonomy, changes in living arrangements, academic demands, and evolving social networks can influence health-related behaviours, including dietary practices [1,2,3]. Because behaviours established during emerging adulthood may persist into later life, universities represent important settings for health promotion and the prevention of non-communicable diseases [4].

Dietary practices are of particular concern among university students. Insufficient consumption of fruits, vegetables, legumes, and other nutrient-dense foods, together with frequent consumption of ultra-processed foods, energy-dense products, and sugar-sweetened beverages, have been widely reported [3,5,6]. These patterns may be influenced not only by individual preferences but also by food availability, affordability, time constraints, food insecurity, cooking skills, social norms, and the university food environment [7,8,9,10,11]. Such influences suggest that dietary behaviour is embedded within broader social and environmental contexts rather than determined exclusively by individual choice.

Research on dietary behaviour among university students has traditionally adopted variable-centred approaches, focusing on overall diet quality or on individual food groups separately. Although overall diet-quality scores provide a useful summary of dietary adherence, they may obscure heterogeneity in the combinations of food-group consumption that characterise individuals. Person-centred approaches provide a complementary perspective by identifying subgroups of individuals with similar multidimensional configurations of food consumption rather than examining each variable independently [12]. In the present study, this perspective was applied to food consumption using the nine frequency-based food-group components of the Healthy Eating Index adapted for the Spanish population (Índice de Alimentación Saludable para la población Española; IASE) [13]. This approach enabled the characterisation of empirically derived configurations of adherence across multiple food groups. Although person-centred methods have been applied to co-occurring lifestyle behaviours among young people and university students [14,15,16,17,18], comparatively little attention has been given to empirically derived food-consumption profiles across university populations from different national settings. This question is particularly relevant in the post-pandemic university environment, where changes in food environments, academic routines, and opportunities for physical activity (PA) have persisted in some settings [7,19].

Beyond its implications for health, food consumption is increasingly considered within a broader sustainability context. Dietary patterns characterised by a greater emphasis on plant-based foods have generally been associated with lower environmental impacts [20], informing frameworks such as the EAT–Lancet Planetary Health Diet [21] and the Planetary Health Diet Index (PHDI) [22]. Several of the food groups considered within these frameworks are also represented in the IASE, highlighting the broader relevance of examining food consumption across multiple food groups.

The present study addresses these gaps using a multicountry sample of university students from Chile, Mexico, Spain, and Italy. Food-consumption profiles were then examined in relation to sociodemographic, academic, anthropometric, and lifestyle characteristics. The present analysis also differs from our previous study using the same dataset, in which CART and CHAID were used to identify subgroups at greater risk of poor diet quality based on the overall IASE score [23]. Whereas that study focused on risk stratification according to a global diet-quality indicator, the present study uses the individual IASE food-group components to identify empirically derived configurations of food consumption. Thus, the current approach seeks to characterise heterogeneity in food consumption rather than to classify participants according to their level of overall diet quality.

This multicountry study aimed to identify food-consumption profiles among university students from Chile, Mexico, Spain, and Italy and to explore the hierarchical associations between profile membership and sociodemographic, academic, anthropometric, and lifestyle characteristics within the participating national settings. By combining person-centred clustering with exploratory segmentation, the study complements conventional analyses based on overall diet-quality scores and provides a multidimensional description of food-consumption heterogeneity across university settings.

## 2. Materials and Methods

### 2.1. Research Design and Participants

A cross-sectional analytical study was conducted to identify food-consumption profiles among university students and to explore the sociodemographic, academic, anthropometric, and lifestyle characteristics associated with profile membership.

The study involved four urban higher-education institutions, one in each participating country: Spain, Mexico, Italy, and Chile. The participating institutions differed in their academic programmes and student populations. Consequently, the study was not designed to achieve institutional or disciplinary equivalence across countries, and the academic composition of each national subsample reflected the programmes available at the participating institution. All eligible students could participate regardless of degree programme. Academic programme was therefore considered a characteristic of the recruited sample rather than a sampling stratum.

For descriptive purposes, the participating degree programmes were grouped into three broad categories: Education-related programmes (*n* = 570; 83.1%), Psychology (*n* = 67; 9.8%), and Other programmes (*n* = 49; 7.1%). The latter category comprised Social Communication, Social Education, and Philosophy. These groupings were used solely to describe the academic composition of the sample and were not used as sampling strata or analytical constraints.

Eligibility criteria were enrolment as an undergraduate student at the participating institution and provision of informed consent. A total of 808 questionnaires were initially collected. Four exclusion criteria were applied. Records were excluded if any of the nine IASE food-group components was missing, since complete data were required to derive the profiles; if the IPAQ-SF could not be scored according to the standard algorithm; if a duplicate submission was identified through submission timestamps and IP information (Section 2.3); or if responses contained logically incompatible values across key variables. After this screening, 122 records (15.1%) were excluded and the final analytical sample comprised 686 students: Chile (*n* = 161; 23.5%), Mexico (*n* = 162; 23.6%), Spain (*n* = 180; 26.2%), and Italy (*n* = 183; 26.7%). Residual missing data in the retained records affected less than 2% of values and were handled as described in Section 2.3.

The study was conducted and reported according to the STROBE guidelines for cross-sectional studies [24].

### 2.2. Data Collection Instruments

Data were collected using a self-administered online questionnaire developed with Google Forms (Google LLC, Mountain View, CA, USA), originally developed in Spanish and translated into Italian using a forward–backward translation procedure involving bilingual experts to preserve semantic and contextual equivalence.

#### 2.2.1. Sociodemographic and Contextual Variables

Participants reported age, country, academic programme, year of study, employment status, living arrangement, perceived financial autonomy, and perceived socioeconomic status. Sex was assessed using a binary response option (“male”/“female”) and was used to describe the sex composition of the sample.

Perceived socioeconomic status was assessed using a single self-rated item in which participants classified their socioeconomic position as low, medium-low, medium, medium-high, or high, with an additional “don’t know/no response” option. This measure captured participants’ subjective perception of their socioeconomic position and was not intended to represent an objective or composite socioeconomic index.

Country was included as a contextual variable reflecting the setting in which participants were recruited. Because each country was represented by a single institution and the institutional and academic composition differed between countries, country was not interpreted as a direct measure of national culture or as an independent national effect.

#### 2.2.2. Health-Related Variables

Participants self-reported height and weight, which were used to calculate body mass index (BMI) and classify weight status according to WHO criteria [25]. Additional variables included current medical or psychological treatment, adherence to a prescribed diet, previous COVID-19 diagnosis, perceived PA restrictions during the pandemic, and self-reported change in PA since the pandemic (higher, lower, or similar to pre-pandemic levels). This latter variable reflected participants’ subjective perception of change and was not treated as a longitudinal measure of behavioural trajectory.

#### 2.2.3. Habitual Physical Activity

Habitual PA was assessed using the IPAQ-SF [26], which evaluates the frequency and duration of walking, moderate-intensity PA, and vigorous-intensity PA performed during the previous seven days.

Weekly PA volume was expressed as metabolic equivalent minutes per week (MET-min/week) according to the standard IPAQ-SF scoring protocol [26,27]. MET-min/week is a composite measure that combines the reported frequency (days/week) and duration (minutes/day) of PA with the corresponding metabolic equivalent (MET) value assigned to each intensity. It therefore represents a weighted volume score rather than an absolute number of minutes of PA. Total weekly PA was calculated by summing the MET-min/week values for walking, moderate-intensity, and vigorous-intensity activities.

Participants were classified into low, moderate or high PA using the standard IPAQ-SF categorical algorithm, which combines the number of days, the daily duration and the intensity of activity with the accumulated weekly volume [26,27]; the criteria defining each category are reported in the note to Table 1.

These categories were retained as the IPAQ-SF classification for the present analyses and were not considered equivalent to the WHO 2020 PA recommendations [28], which recommend 150–300 min/week of moderate-intensity aerobic PA, 75–150 min/week of vigorous-intensity aerobic PA, or an equivalent combination. Thus, the two frameworks are related but not equivalent.

Given the established use and reliability of the IPAQ-SF [27,29] and the recognised limitations of self-reported PA [30], the resulting measures were interpreted as behavioural indicators rather than objective estimates of energy expenditure. They were subsequently used as explanatory characteristics in the comparative and segmentation analyses.

#### 2.2.4. Food Consumption

A person-centred clustering approach was used to identify subgroups of participants characterised by similar multidimensional profiles of food-group consumption.

Food consumption was assessed using the Healthy Eating Index adapted for the Spanish population (Índice de Alimentación Saludable para la población Española; IASE), developed by Norte and Ortiz [13] as an adaptation of the original Healthy Eating Index developed by the U.S. Department of Agriculture in 1995 (HEI-1995) [31] to Spanish dietary recommendations. The IASE assesses adherence to recommended consumption frequencies across major food groups rather than nutrient intake. Accordingly, it provides a food-based indicator of dietary adherence rather than a comprehensive measure of nutrient intake or overall dietary composition.

In the present study, dietary profiles were derived from the nine frequency-based IASE component scores, rather than from predefined dietary categories or the overall IASE score. The IASE was used as a common food-based framework to characterise dietary behaviour among participants from Chile, Mexico, Spain, and Italy. Its purpose was therefore to provide a harmonised basis for cross-national comparison, rather than to assess compliance with the specific dietary guidelines of each participating country. IASE scores should consequently be interpreted as harmonised indicators of adherence to the food-group recommendations incorporated into the IASE and not as country-specific measures of compliance with national dietary guidelines.

The Spanish origin of the instrument should also be considered when interpreting the findings. Differences in national dietary recommendations, food cultures, and customary consumption patterns may not be fully captured by the IASE framework. Formal cross-cultural measurement invariance was not assessed; therefore, potential differences in the functioning of the IASE component scores across the four national settings cannot be excluded and should be considered when interpreting cross-national comparisons.

The original IASE comprises ten food-consumption components, including dietary variety. The nine frequency-based components are scored using a common five-level adherence scale ranging from 0 to 10 (0, 2.5, 5.0, 7.5, and 10), whereas dietary variety is scored separately on a 0–2 scale. Dietary variety was therefore excluded from the clustering procedure because it is assessed using a different metric. The resulting profiles were derived exclusively from the nine frequency-based components, all expressed on the same 0–10 scale. Higher scores consistently indicate greater adherence to the recommendation for the corresponding food group; depending on the food group, this may reflect either more frequent consumption of foods recommended for regular intake or less frequent consumption of foods for which intake is recommended to be limited.

The IASE and other HEI-derived indices have previously been used to characterise diet quality and differentiate dietary behaviours among university students [32,33]. The use of the nine individual component scores, rather than the overall IASE score, preserved the multidimensional structure of food-group adherence and enabled participants with similar configurations across components to be grouped into distinct profiles. These profiles should therefore be interpreted as configurations of adherence to food-group consumption recommendations rather than as dietary patterns in the conventional nutritional-epidemiology sense, as the IASE does not capture portion sizes, nutrient intake, or meal timing.

### 2.3. Procedures and Ethical Considerations

Data collection was conducted from November 2023 to April 2024. The questionnaire was distributed electronically through institutional channels, including email, virtual learning environments, and classroom announcements, using standardised information and instructions provided by teaching staff. Participation was voluntary and anonymous, and no incentives were provided. Before completing the questionnaire, participants received information about the study objectives and requirements, confidentiality and data-protection procedures.

All participants were enrolled undergraduate students who provided their own informed consent electronically. The recruitment and consent procedures, including the eligibility of the student population approached, were approved by the Ethics Committee of the Institute of Agricultural and Rural Sciences (ICAR) of the Universidad Autónoma del Estado de México (UAEMéx) (approval code: 01-SEP-2023; approved 14 July 2023), and no additional consent from a legal representative was required for participation.

The questionnaire required approximately 12 min to complete. Responses were stored securely, and potential duplicate submissions were screened using submission timestamps and IP information. Missing data accounted for less than 2% of the dataset and were handled using pairwise deletion. The study was conducted in accordance with the ethical principles of the Declaration of Helsinki [34].

### 2.4. Data Processing and Statistical Analysis

Analyses were performed using IBM SPSS Statistics for Windows, version 29.0 (IBM Corp., Armonk, NY, USA), with statistical significance set at *p* < 0.05 (two-tailed). Data were screened for missing values and implausible observations before analysis. Continuous variables were summarised as mean ± standard deviation (SD) or median and interquartile range (IQR), as appropriate. Distributional characteristics were assessed using Shapiro–Wilk tests together with histogram and Q–Q plot inspection, while Levene’s test was used to assess homogeneity of variance where relevant. Because several variables showed departures from normality and/or heterogeneity of variance, non-parametric procedures were used as the primary approach for group comparisons.

#### 2.4.1. Preliminary Analyses

Descriptive analyses were conducted for the overall sample and separately by country. Associations between categorical variables were examined using Pearson’s chi-square test, with Cramer’s V used as an effect-size measure and adjusted standardised residuals used to identify cells contributing to significant associations. Continuous and ordinal variables were compared using Kruskal–Wallis tests, followed, where appropriate, by Dunn post hoc pairwise comparisons with Bonferroni adjustment for the number of pairwise contrasts, for which adjusted significance values are reported. These preliminary analyses were used to characterise the sample and examine differences across countries and subsequently identified food-consumption profiles.

#### 2.4.2. Identification of Food-Consumption Profiles

The analytical strategy comprised two sequential but conceptually distinct stages. First, hierarchical clustering followed by k-means refinement was used to identify food-consumption profiles from the nine IASE component scores. Second, the resulting profile-membership variable was used as the outcome in an exploratory Exhaustive CHAID analysis to examine how sociodemographic, academic, anthropometric, and lifestyle characteristics were hierarchically associated with profile membership. Clustering addressed which food-consumption configurations were present in the sample, whereas CHAID examined the characteristics associated with membership in those configurations.

Food-consumption profiles were identified through unsupervised clustering of the nine IASE component scores (see Section 2.2.4), rather than from predefined dietary categories or the overall IASE score. Although these scores share a common five-level 0–10 metric, their dispersion differed appreciably across food groups (variances ranging from 4.78 for vegetables and greens to 11.10 for sugar-sweetened beverages). Because Euclidean distance weights each variable in proportion to its variance, the components with the widest dispersion would otherwise have dominated the definition of the clusters, whereas components with adherence concentrated towards the upper end of the scale would have contributed little. The nine component scores were therefore standardised (z-scores) before clustering, so that adherence to each food-group recommendation contributed equally to the distance between participants. This decision reflects the configurational aim of the analysis: to characterise the pattern of adherence across all nine food groups rather than the ranking of participants on the most heterogeneous ones. Its consequences for cluster stability are reported in Section 3.4 and considered in Section 4.5.

A two-stage clustering strategy was used. Hierarchical clustering with Ward’s method and squared Euclidean distance was first applied to solutions ranging from two to five clusters. K-means refinement was then performed using the hierarchical cluster centres as initial seeds. Candidate solutions were evaluated using the Calinski–Harabasz index, Davies–Bouldin index, and silhouette coefficient, criteria commonly used to evaluate the internal validity of empirically derived dietary patterns [35], together with split-half stability testing [36]. Because no candidate solution consistently outperformed the alternatives across all quantitative criteria (see Section 3.4), the four-cluster solution was retained for the primary exploratory analysis on the basis of its combined quantitative adequacy, subgroup size, and substantive interpretability. This selection was therefore not based on a single statistical criterion; accordingly, the four-cluster solution should be regarded as exploratory and requiring replication.

Split-half stability was assessed across 100 random 50/50 partitions. Within each partition, each half was independently clustered using the same hierarchical clustering and k-means refinement procedure. Cases in each half were then assigned to the cluster centroids derived from the opposite half using the nearest-centroid rule. Agreement between the native clustering solution and the classification obtained from the opposite half’s centroids was quantified using the Adjusted Rand Index (ARI), Cramer’s V, and the misclassification rate after optimal cluster-label alignment using the Hungarian algorithm. The same validation procedure was applied to the two-, three-, four-, and five-cluster solutions.

Because the validation criteria did not identify a uniformly superior solution, the substantive distinctiveness of the four-cluster solution was further examined by comparing it with the three-cluster solution in relation to the sociodemographic, academic, anthropometric, and lifestyle characteristics described in Section 2.4.3. These post hoc sensitivity analyses used chi-square tests with adjusted standardised residuals and Cramer’s V, applying the Benjamini–Hochberg false discovery rate correction for multiple comparisons. The IASE component scores themselves were used to characterise the profiles rather than as inferential comparison variables because they constituted the variables used to generate the clusters.

Profiles were subsequently labelled according to their characteristic IASE component configurations. Labels were assigned post hoc to facilitate interpretation and were not generated by the clustering procedure. In particular, the label “Protein-Oriented” refers primarily to the profile’s relative configuration on the IASE meat, fish, and eggs component, which reflects adherence to the recommended consumption frequency for this food group. It should therefore not be interpreted as indicating the highest absolute protein intake or the highest overall consumption of animal-source foods.

#### 2.4.3. Characterisation of Food-Consumption Profiles

Differences in food-consumption profile membership were examined according to country, sex, age, academic programme, year of study, perceived socioeconomic status, living arrangement, employment status, perceived financial situation, BMI, BMI-derived weight status according to WHO criteria, adherence to a prescribed diet, current medical or psychological treatment, COVID-19 history, perceived pandemic-related PA restrictions, self-reported change in PA since the pandemic, and habitual PA level. Categorical variables were compared using Pearson’s chi-square tests, with adjusted standardised residuals used to identify cells contributing to significant associations and Cramer’s V reported as an effect-size measure. Continuous and ordinal variables were compared using Kruskal–Wallis tests, followed, where appropriate, by Dunn post hoc pairwise comparisons with Bonferroni adjustment for the number of pairwise contrasts, for which adjusted significance values are reported.

Because 17 participant characteristics were examined in parallel, the resulting *p*-values were corrected for multiple comparisons using the Benjamini–Hochberg false discovery rate procedure, applied jointly to the full family of 17 tests (15 chi-square tests and two Kruskal–Wallis tests) at a false discovery rate of 0.05. Both raw and adjusted *p*-values are reported in Supplementary Table S3, and associations described as significant in Section 3.3 are those that remained significant after this correction. Adjusted standardised residuals were subsequently inspected only for variables that survived the correction; they were interpreted at |z| ≥ 1.96 without further adjustment and are therefore exploratory indications of the cells contributing to each association.

#### 2.4.4. Hierarchical Segmentation Analysis

An Exhaustive CHAID analysis was conducted with food-consumption profile membership as the dependent variable and the same sociodemographic, academic, anthropometric, and lifestyle variables used for profile characterisation as candidate predictors. This recursive-partitioning procedure was selected because it can accommodate categorical and continuous predictors and identify hierarchical combinations of characteristics associated with a categorical outcome without requiring a prespecified linear functional form [37]. The resulting tree therefore provided an exploratory representation of how profile membership was associated with combinations of participant characteristics.

Tree growth used Bonferroni-adjusted chi-square tests, a maximum depth of four levels, minimum parent and child node sizes of 60 and 30 cases, respectively, and ten-fold cross-validation. These parameters were specified to maintain interpretable subgroup sizes and limit excessive tree complexity [37,38]. The resulting tree was interpreted as an exploratory representation of hierarchical associations with food-consumption profile membership rather than as a predictive classification model. Because the analysis was exploratory, the observed splits were interpreted as sample-specific associations requiring replication rather than as evidence of causal relationships or generalisable prediction.

## 3. Results

### 3.1. Sample Characteristics

The study included 686 university students from four countries, with a relatively balanced distribution across national subsamples: Chile (23.5%), Mexico (23.6%), Spain (26.2%), and Italy (26.7%) (Table 1). Median age was 21.3 years (IQR: 19.49–23.31), and 60.8% of participants were women. Most students were enrolled in Education-related programmes (83.1%), followed by Psychology (9.8%) and Other programmes (7.1%). The latter category comprised Social Communication, Social Education, and Philosophy.

Approximately half of the sample perceived their socioeconomic status as medium (49.1%). Most participants lived with close family members (83.8%), were unemployed (61.7%), and were fully financially dependent (59.2%). Median BMI was 22.98 kg/m^2^ (IQR: 20.80–25.50); 62.6% of participants were classified as having normal weight, 25.0% as overweight, and 5.4% as having obesity. Only 7.4% reported following a medically prescribed diet, while 81.0% reported no current medical or psychological treatment.

Regarding COVID-19 history, 50.3% reported no known previous infection and 30.8% reported a diagnosis by a healthcare professional. Overall, 39.8% reported no physical-activity restriction during the pandemic, whereas the remainder reported restrictions attributed to COVID-19-related physical consequences, other causes, or lockdown and space restrictions. After the pandemic, 42.3% perceived themselves as more physically active than before, whereas 27.4% reported lower PA levels. According to the standard IPAQ-SF classification algorithm, 59.3% of participants were classified as having high PA, 24.2% as moderate, and 16.5% as low PA.

### 3.2. Identification and Characterisation of Food-Consumption Profiles

**Figure 1:**
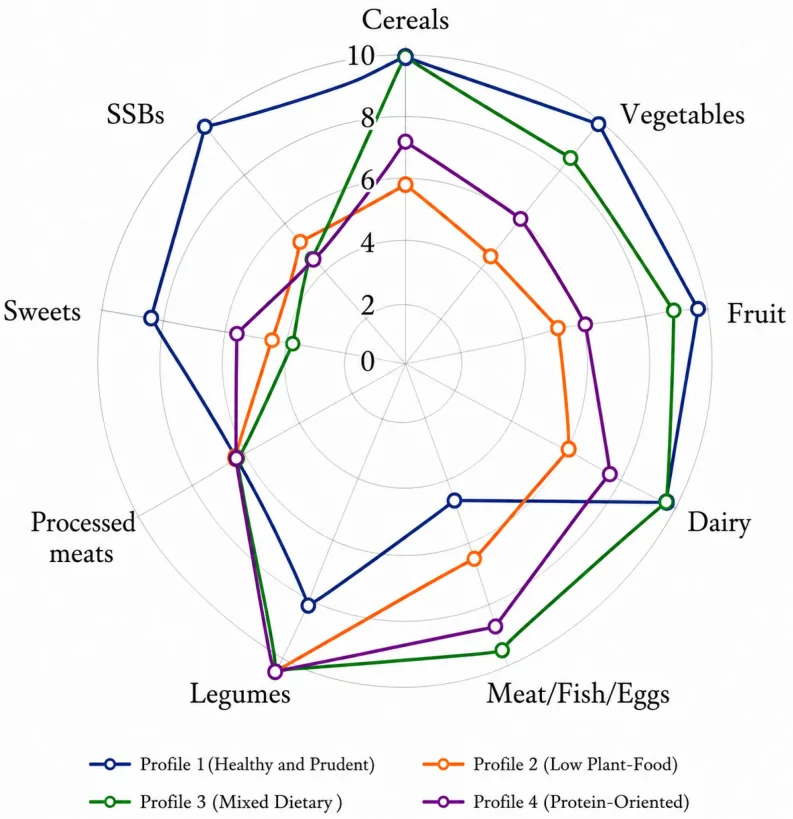
Radar plot illustrating the dietary configuration of the four food-consumption profiles identified using IASE component scores. Values represent the median IASE component scores (0–10) for each profile. Higher scores indicate greater adherence to the corresponding IASE recommendation. For processed meats and cold cuts, sweets, and sugar-sweetened beverages (SSBs), higher scores indicate lower consumption and therefore greater adherence to the relevant recommendation.

Cluster analysis based on the nine IASE component scores identified four food-consumption profiles, each representing a distinct configuration of adherence to the food-group recommendations operationalised by the IASE. Median component scores are presented in Table 2, and the corresponding configurations are illustrated in Figure 1.

The four profiles differed mainly in their relative adherence to the recommended consumption frequencies for plant-based foods, meat/fish/eggs, sweets and sugar-sweetened beverages, the greatest differentiation being observed for legumes, fruit, vegetables, sugar-sweetened beverages and meat/fish/eggs (Table 2; Figure 1). For ease of presentation, they were labelled Healthy and Prudent (Profile 1), Low Plant-Food (Profile 2), Mixed Dietary (Profile 3) and Protein-Oriented (Profile 4). These labels are descriptive shorthand summarising the most prominent features of each configuration of IASE component scores; they were not generated by the clustering algorithm and should not be read as evaluative rankings.

These four clusters should not be interpreted as a validated dietary typology. As detailed in Section 3.4, internal validity indices did not favour the four-cluster solution over the two-, three-, or five-cluster alternatives (silhouette coefficients 0.113–0.126 for all candidate solutions), split-half stability was limited throughout (mean Adjusted Rand Index 0.10–0.35, including 0.30 for the four-cluster solution), and Profile 4 does not survive as a distinct configuration under the three-cluster solution. Among the characteristics subsequently examined (Section 3.3), country and academic programme—themselves confounded with participating institution (Section 4.5)—showed the largest, although still modest, association with profile membership (Cramer’s V = 0.165–0.179); no other characteristic approached this magnitude. The profiles described below should accordingly be read as an exploratory, sample-specific configuration rather than an established or generalisable dietary typology.

The Healthy and Prudent profile showed comparatively high adherence across several components, particularly fruit, vegetables, cereals and legumes, together with relatively favourable scores for the foods recommended to be limited. The Low Plant-Food profile was characterised primarily by lower adherence for fruit and legumes and by lower vegetable scores than the other profiles, with intermediate scores across most remaining components. The Mixed Dietary profile combined relatively high adherence to both plant-based food groups and the meat/fish/eggs component, together with high adherence to dairy products, resulting in a heterogeneous rather than a uniformly plant-oriented configuration. The Protein-Oriented profile showed relatively high scores for legumes and meat/fish/eggs, the latter being the most prominent feature of its configuration, and intermediate scores for most other components.

To facilitate interpretation of the profiles independently of the 0–10 IASE adherence transformation, the underlying food-group frequencies were additionally summarised using their original response scale (0 = never/almost never to 4 = daily). Plant-based and animal-source food groups were aggregated into descriptive composites and compared across the four profiles (Table 3). This analysis was intended to aid interpretation of the cluster configurations rather than to provide an independent validation of the clustering solution, because the same underlying food-group variables contributed to profile derivation.

Profile 2 showed the lowest mean frequency of consumption of the food groups included in the plant-based composite (mean *=* 2.24, SD = 0.86), consistent with its Low Plant-Food designation. Profile 3, the largest profile, showed the highest mean frequencies for both the plant-based composite (mean = 3.23, SD = 0.38) and the animal-source composite (mean = 2.99, SD = 0.61), indicating relatively frequent consumption across both sets of food groups rather than a predominantly plant-based configuration. Profile 1 showed a similarly high plant-based frequency (mean = 3.21, SD = 0.42), whereas Profile 4 had a lower plant-based frequency (mean = 2.30, SD = 0.43).

Differences between profiles were statistically significant for both composites, with a large effect for the plant-based composite (H(3) = 327.50, *p* < 0.001, ε^2^ = 0.476) and a small effect for the animal-source composite (H(3) = 70.42, *p* < 0.001, ε^2^ = 0.099). These comparisons should nevertheless be interpreted descriptively because the component frequencies underlying the composites were also represented, after transformation into IASE adherence scores, among the variables used to derive the clusters.

### 3.3. Sociodemographic, Anthropometric and Lifestyle Characteristics According to Food-Consumption Profiles

Significant differences between food-consumption profiles were observed for age, country, academic programme, year of study, habitual PA level, medically prescribed diet, and self-reported PA change following the pandemic (Table 4). Effect sizes were small, ranging from small: ε^2^ = 0.039 for age, and Cramer’s V values up to 0.179 for academic programme. No significant differences were observed for sex, perceived socioeconomic status, living arrangement, employment status, financial situation, BMI category, current medical or psychological treatment, COVID-19 history, or perceived restrictions on PA during the pandemic (Table 4).

Country was significantly associated with profile membership (χ^2^(9) = 55.81, *p* < 0.001, Cramer’s V = 0.165). Table 4 reports within-profile percentages. The comparisons in this paragraph are instead expressed as within-country percentages, that is, as the share of each national subsample assigned to a given profile; elsewhere in this section percentages follow Table 4. The Healthy and Prudent profile accounted for a larger share of the Italian subsample (34.4% of Italian students) than of the Spanish (29.4%), Chilean (17.4%) or Mexican (16.0%) subsamples. The Mixed Dietary profile was the most frequent configuration in Chile (45.3%) and Mexico (40.7%) and the least represented in Italy (29.5%). The Protein-Oriented profile was most frequent in Mexico (32.7%) and Spain (31.1%), which differed little from each other, and least frequent in Italy (14.2%). The Low Plant-Food profile was most frequent in Italy (21.9%) and least frequent in Spain (7.2%). The four profiles were therefore unevenly distributed within the Chilean, Mexican and Italian subsamples, whereas in Spain three of them were of comparable size (29.4–32.2%) and only the Low Plant-Food profile was clearly smaller. Academic programme showed the largest categorical effect size (χ^2^(21) = 66.09, *p* < 0.001, Cramer’s V = 0.179). This association should be interpreted with caution: academic programme is almost entirely nested within institution and country, and is therefore largely redundant with country rather than indicative of a disciplinary effect.

Age also differed between profiles, although the magnitude of the association was small (H(3) = 29.83, *p* < 0.001, ε^2^ = 0.039). Post hoc Dunn comparisons with Bonferroni adjustment for the six pairwise contrasts indicated that participants in the Protein-Oriented profile were younger than those in the Healthy and Prudent (standardised test statistic = 4.78, adjusted *p* < 0.001), Low Plant-Food (4.29, adjusted *p* < 0.001) and Mixed Dietary (2.72, adjusted *p* = 0.039) profiles. No other pairwise comparison reached significance after adjustment.

Habitual PA level was associated with profile membership (Cramer’s V = 0.143). The Low Plant-Food profile included the highest proportion of participants classified as having low PA (33.7%), whereas high PA was most frequent in the Protein-Oriented (64.2%) and Healthy and Prudent (62.4%) profiles, followed by the Mixed Dietary profile (60.2%). Self-reported change in PA following the pandemic was also associated with profile membership, although the effect was small (Cramer’s V = 0.114). The proportion of participants reporting having become more physically active was highest in the Mixed Dietary profile (26.7%, compared with 20.6%, 19.9% and 13.5% in the Healthy and Prudent, Protein-Oriented and Low Plant-Food profiles, respectively), whereas reduced PA (less or much less active) was most frequent in the Low Plant-Food profile (41.6%). The Healthy and Prudent profile had the highest proportion of participants reporting no change in PA (37.1%).

A medically prescribed diet was also associated with profile membership (Cramer’s V = 0.165). The proportion reporting a prescribed diet was highest in the Healthy and Prudent profile (14.7%) and substantially lower in the Mixed Dietary (4.4%) and Protein-Oriented (4.5%) profiles. This association was considered descriptive because the study design does not establish whether following a prescribed diet preceded or resulted from the observed food-consumption configuration.

The bivariate analyses therefore associated profile membership primarily with academic programme and country, followed by medically prescribed diet, year of study, habitual PA level, self-reported PA change and age. The remaining sociodemographic, anthropometric, health-related and pandemic-related variables showed no significant differences after correction for multiple comparisons. These associations were subsequently examined using Exhaustive CHAID to determine whether they formed interpretable hierarchical combinations within the multicountry sample.

### 3.4. Sensitivity Analyses of the Four-Cluster Solution

The internal validity and stability analyses did not identify a cluster solution with adequate performance across the quantitative criteria (Supplementary Table S1). The two-cluster solution yielded the highest Calinski–Harabasz index (90.1) and the three-cluster solution the highest silhouette coefficient (0.126), whereas the five-cluster solution yielded the lowest Davies–Bouldin index (1.916) together with the highest split-half stability (mean Adjusted Rand Index [ARI] = 0.35; mean Cramer’s V = 0.59; mean misclassification rate = 40.6%). Silhouette coefficients were low for all candidate solutions (range 0.113–0.126), indicating limited separation and substantial overlap between profiles. The retained four-cluster solution was not superior on any individual criterion (Calinski–Harabasz = 80.9; Davies–Bouldin = 2.072; silhouette = 0.113) and showed limited stability (mean ARI = 0.30; mean Cramer’s V = 0.58; mean misclassification rate = 40.2%). Split-half stability was low throughout (mean ARI between 0.10 and 0.35), indicating that the boundaries between profiles were not consistently reproduced across random subsamples. The quantitative criteria therefore did not support the selection of any particular number of clusters.

Because the four-cluster solution was retained for the primary exploratory analysis, its relationship with the three-cluster solution was further examined. Under the three-cluster solution, Profile 4 was not retained as a separate configuration: 83.5% of its members were assigned to the cluster corresponding predominantly to Profile 3, and 15.9% to the cluster corresponding predominantly to Profile 1 (Supplementary Table S2). Profile 4 therefore behaved largely as a subdivision of a single broader cluster under the more parsimonious partition, and its retention as a separate profile is not supported by this comparison. Its distinctiveness was consequently examined against the full set of characterisation variables, as described below.

The substantive characteristics of Profile 4 were subsequently examined using the full set of variables included in profile characterisation. After Benjamini–Hochberg correction, seven variables remained significantly associated with overall profile membership: age, country, academic programme, year of study, habitual PA level, medically prescribed diet, and self-reported PA change (Supplementary Table S3). Examination of adjusted standardised residuals indicated that Profile 4 was specifically differentiated by country, academic programme, and year of study. Profile 4 was overrepresented among Mexican students and underrepresented among Italian students (adjusted residuals = 2.35 and −4.14; χ^2^(9) = 55.81, *p* < 0.001). It was also overrepresented among Pedagogy students and underrepresented among Clinical Psychology and Educational Psychology students (3.33, −2.12 and −3.03; χ^2^(21) = 66.09, *p* < 0.001). Finally, it was overrepresented among first-year students and underrepresented among fourth-year students (4.04 and −2.90; χ^2^(12) = 45.91, *p* < 0.001). Although habitual PA level, medically prescribed diet, and self-reported PA change were also associated with overall profile membership, no adjusted residual involving Profile 4 reached statistical significance for these variables.

None of the candidate solutions was statistically superior, and stability was limited across all of them, with the five-cluster solution showing the highest values. The four-cluster solution was nonetheless retained because it yielded four subgroups of sufficient size (*n =* 170, 89, 251, and 176), a substantively interpretable configuration of food-group adherence, and a profile (Profile 4) whose membership showed specific associations with country, academic programme, and year of study. The internal validity and stability indices did not support this choice, which should therefore be regarded as exploratory and requiring replication in independent samples [35].

Because the purpose of the clustering stage was to identify food-consumption configurations, the subsequent Exhaustive CHAID analysis was conducted separately to examine how the characteristics associated with profile membership were hierarchically organised within the selected four-profile solution.

### 3.5. Multivariate Segmentation Analysis (CHAID)

**Figure 2:**
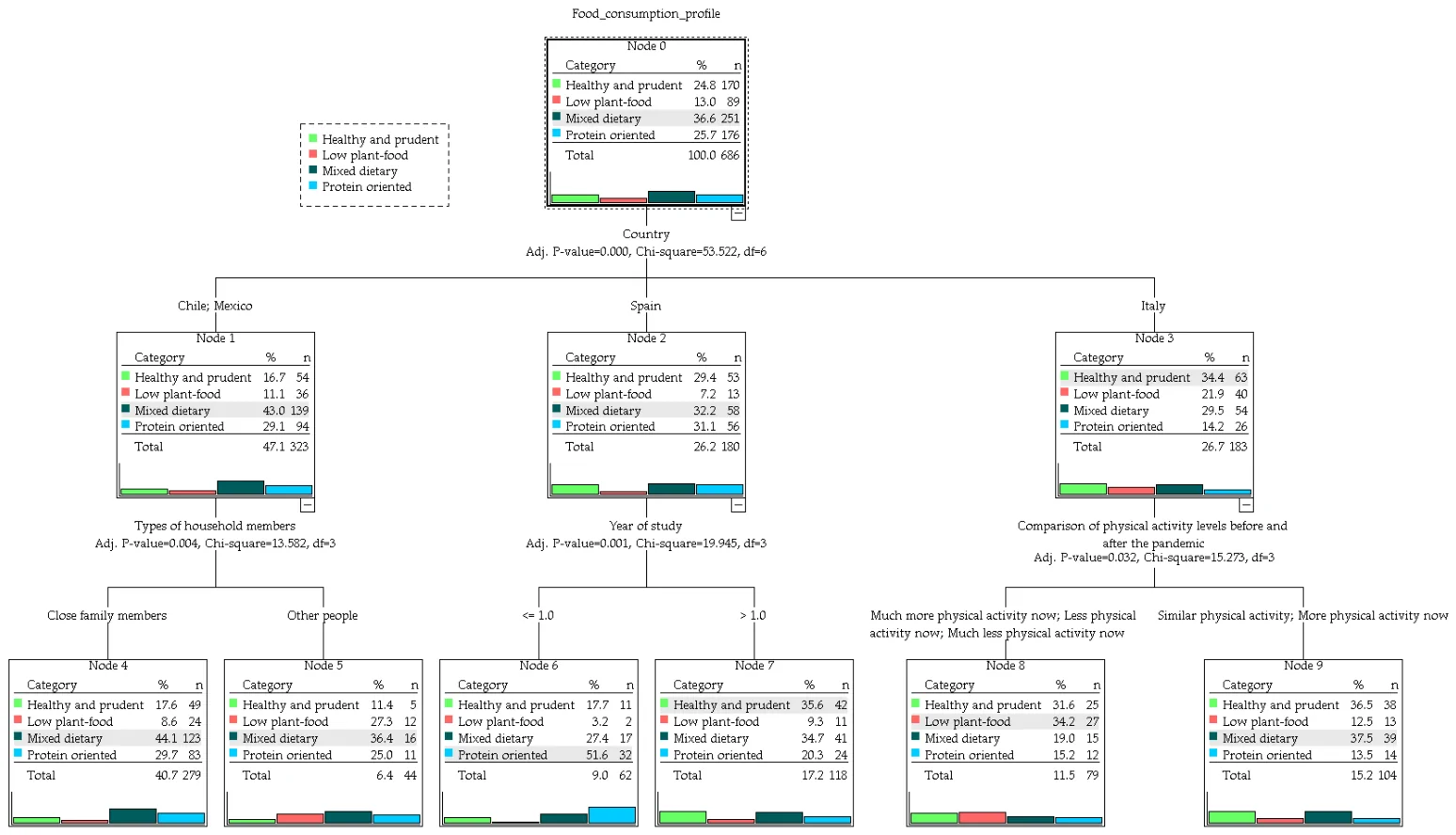
Exhaustive CHAID decision tree showing the hierarchical segmentation of food-consumption profiles according to sociodemographic and lifestyle characteristics. The tree was grown using the Exhaustive CHAID algorithm with chi-square tests and Bonferroni-adjusted significance levels for node splitting. The maximum tree depth was set at four levels, with minimum parent and child node sizes of 60 and 30 cases, respectively. Ten-fold cross-validation was applied to assess model stability. Terminal nodes display the distribution of the four food-consumption profiles within each subgroup, with the predominant profile indicated by the highest percentage.

To further characterise the identified food-consumption profiles, an Exhaustive CHAID analysis was performed using profile membership as the dependent variable and the selected sociodemographic, academic, anthropometric, and lifestyle variables as candidate predictors. The resulting decision tree is presented in Figure 2.

Among the variables entered into the model, only country, living arrangement, year of study, and self-reported change in PA following the pandemic were retained in the final tree. Country constituted the first split (χ^2^ = 53.522, *df* = 6, Bonferroni-adjusted *p* < 0.001), separating participants into Chile/Mexico, Spain, and Italy. Within the Chile/Mexico branch, living arrangement provided the subsequent split (χ^2^ = 13.582, *df* = 3, Bonferroni-adjusted *p* = 0.004). Among Spanish students, the next split was based on year of study (χ^2^ = 19.945, *df* = 3, Bonferroni-adjusted *p* = 0.001), whereas self-reported change in PA following the pandemic provided the subsequent split within the Italian branch (χ^2^ = 15.273, *df* = 3, Bonferroni-adjusted *p* = 0.032).

The final tree comprised 10 nodes, including six terminal nodes, and reached an observed depth of two levels despite allowing a maximum depth of four. The cross-validated risk estimate was 0.650 (SE = 0.018), compared with 0.593 (SE = 0.019) for resubstitution and with 0.634 for the majority-class baseline, obtained by assigning all participants to the largest profile (Mixed Dietary; 251/686). The tree therefore did not improve on the baseline in raw classification accuracy, and the small difference between resubstitution and cross-validation risk indicates limited overfitting rather than predictive performance.

Inspection of the terminal nodes showed different profile distributions according to the combinations of characteristics identified within each national branch. Among students from Chile and Mexico living with close family members, the Mixed Dietary profile was predominant (44.1%; Node 4) and remained the most represented profile among those living with people other than close family members (36.4%; Node 5), although the Low Plant-Food profile rose from 8.6% to 27.3% between the two nodes. Among Spanish students, the Protein-Oriented profile was particularly frequent among those in their first year of study (51.6%; Node 6), whereas the Healthy and Prudent profile predominated among students in later years (35.6%; Node 7). Within the Italian subsample, the Mixed Dietary profile was the most frequently represented among students reporting similar or moderately increased PA after the pandemic (37.5%; Node 9), whereas the Low Plant-Food profile was more represented among those reporting either reduced PA or a marked increase (34.2%; Node 8). Node 9 comprised participants reporting similar or moderately increased PA (*n =* 104); Node 8 comprised those reporting a marked increase or any decrease (*n =* 79). The Exhaustive CHAID algorithm treated this predictor as nominal and therefore permitted the merging of non-adjacent response categories.

The characteristics associated with food-consumption profile membership differed across national branches. Country formed the initial partition, after which different characteristics were retained within each branch: living arrangement in Chile and Mexico, year of study in Spain, and self-reported post-pandemic PA change in Italy. These findings indicate that the associations observed with profile membership were not organised according to a single common segmentation structure across the four participating settings.

These segmentation structures are descriptive associations that require replication in independent and preferably larger samples and, together with the low silhouette coefficients and the limited split-half stability of the clustering solutions, warrant a cautious reading of both analytical stages.

## 4. Discussion

### 4.1. Principal Findings

Before considering these findings, it should be recalled that the four-cluster solution is exploratory: none of the internal validity or split-half stability indices favoured it over the alternative solutions, and Profile 4 does not survive as a distinct configuration under the more parsimonious three-cluster solution (Section 3.4). With this caveat in mind, the present multicountry study identified four food-consumption profiles among university students, with all four profiles represented in Chile, Mexico, Spain, and Italy. The profiles differed in their configurations of adherence to the nine IASE food-group recommendations, indicating that dietary behaviour in this sample was not adequately represented by a single continuum of overall diet quality. The main finding, however, concerns what was observed after profile identification: the characteristics associated with profile membership differed across national contexts. Country constituted the first split in the Exhaustive CHAID analysis, after which living arrangement was retained within the Chile/Mexico branch, year of study within the Spanish branch, and self-reported change in PA following the pandemic within the Italian branch.

These are context-specific statistical associations, not causal pathways, determinants or predictive rules: the analysis indicates which characteristics accompanied the distribution of profiles within each national setting, not which characteristics predict an individual participant’s profile.

The findings complement previous research that has examined overall diet-quality scores, individual dietary components, predefined dietary patterns, or clusters of multiple health behaviours [14,15,16,39,40]. In contrast, the present study derived food-consumption profiles empirically from the multidimensional configuration of nine IASE component scores and subsequently examined their associations with contextual characteristics. Importantly, participant characteristics were not used to construct the profiles. Consequently, the study does not identify combined diet–PA or broader health-behaviour profiles; instead, it identifies dietary configurations and examines whether their distribution is associated with different characteristics within each national context.

Decision-tree methods offer a way of describing how membership in the identified profiles was distributed across combinations of participant characteristics. They have been used to examine hierarchical combinations and interactions in eating-related outcomes [41], and methodological research on classification trees highlights their capacity to generate interpretable decision rules and identify subgroups with distinct characteristics [42]. In the present study, however, CHAID was used neither to establish causal pathways nor to optimise individual-level prediction, but to explore how the identified food-consumption profiles were contextually distributed across combinations of participant characteristics. The results therefore suggest that similar food-consumption configurations may coexist across different university settings without necessarily showing the same pattern of associated characteristics. This distinction may be relevant for interpreting multicountry dietary research, where between-country differences in profile prevalence alone may provide limited information about the circumstances associated with those profiles.

The findings are also consistent with a broader socioecological view of university dietary behaviour, in which food consumption may be shaped by interacting individual, social, and environmental conditions [1,9,43]. However, the present cross-sectional design does not allow these influences to be disentangled or causal relationships to be established. The associations identified here should consequently be considered hypotheses for further investigation rather than evidence of specific contextual mechanisms.

### 4.2. Interpretation of the Identified Food-Consumption Profiles

The four profiles illustrate that food-consumption behaviour among the participating students cannot be adequately summarised by a single healthy–unhealthy continuum. Although the IASE provides a useful summary of adherence to food-group recommendations, the component scores capture different aspects of food consumption that may combine in distinct ways. The person-centred analysis therefore provides complementary information by showing how adherence across food groups was configured within subgroups of students [15,16].

The Healthy and Prudent profile showed relatively high adherence across several food groups, particularly fruit, vegetables, legumes, and the recommendations concerning sugar-sweetened beverages, together with a comparatively lower score for meat, fish, and eggs. The label should therefore be understood as a descriptive summary of its overall configuration rather than as an indication that this profile was uniformly healthier than the others. Its pattern is broadly compatible with evidence from university populations linking healthier dietary practices with more favourable lifestyle and well-being characteristics, including recent findings among Spanish university students showing associations between adherence to the Mediterranean diet and lifestyle habits and mental and emotional well-being [5,44]. This comparison should nevertheless be interpreted cautiously, as the present profiles were derived from IASE food-group component scores and were not designed to measure adherence to the Mediterranean diet.

Although food availability, affordability, time constraints, and food-preparation skills have been identified as relevant individual and contextual factors in university students’ food choices [1,45], food insecurity and time availability have also been associated with diet-related behaviours in university populations [46]. However, the present cross-sectional data did not assess these factors as explanatory mechanisms and therefore cannot establish whether they were related to membership in this profile.

The Mixed Dietary profile was the largest subgroup and combined relatively favourable adherence for several recommended food groups with lower adherence for others. Its configuration illustrates that favourable and less favourable food-consumption characteristics can coexist within the same subgroup rather than necessarily forming opposite ends of a single dietary continuum [15,39]. It should therefore not be interpreted as an intermediate or transitional stage between healthier and less healthy dietary behaviour.

The Protein-Oriented profile was characterised particularly by a relatively higher adherence score for the meat, fish, and eggs component, together with relatively lower scores for cereals, fruit, and vegetables. Protein prioritisation has been reported among university students in the context of weight-, shape-, and body-composition-related goals, including strategies involving increased protein consumption [47]. More broadly, nutritional attitudes, motivation, and body-composition characteristics have been shown to be interrelated among university students [48]. However, the present study did not directly assess these motives, nutritional attitudes, or body-composition goals and therefore cannot establish that they explain the observed configuration.

The additional analysis using raw consumption frequency provided a complementary description of the profiles. The Low Plant-Food profile showed the lowest frequency of consumption of the plant-based food groups included in the composite, whereas the Mixed Dietary profile showed the highest frequencies for both plant-based and animal-source food groups. The latter finding is important because frequent consumption of both types of food does not necessarily correspond to a predominantly plant-based dietary configuration. Accordingly, the observed profiles should not be read as a gradient of dietary sustainability, since environmental impacts were not assessed (Section 4.5).

Comparable levels of adherence to individual food-group recommendations may therefore combine in different ways across students. This configurational heterogeneity is not conveyed by a single summary score, since equivalent totals may arise from distinct arrangements of compliance across food groups. Person-centred analyses therefore complement conventional a priori dietary indices rather than substituting for them [49,50]. Whether this additional information improves the discrimination of health-related outcomes was not tested in the present design and remains an open question.

These profiles should nonetheless be read as configurations of adherence to the IASE food-group recommendations, not as dietary patterns in the broader nutritional-epidemiological sense. That construct encompasses the combination, variety, frequency and quantity of the foods habitually consumed [51]. The IASE assesses neither portion sizes nor nutrient intake, and it disregards the temporal distribution of eating occasions [13]—a dimension shown to carry information independent of overall adherence scores in university students [52].

### 4.3. Cross-National Heterogeneity and Context-Specific Associations

Although the same four food-consumption profiles were identified in Chile, Mexico, Spain, and Italy, their distribution and associated characteristics differed across the participating settings. This finding is more informative than a simple comparison of dietary prevalence between countries because it indicates that similar food-consumption configurations can coexist with different characteristics associated with profile membership. The CHAID analysis provided an exploratory description of these context-specific associations rather than evidence that particular characteristics determine dietary profile membership.

Country was the first variable retained, separating Chile and Mexico from Spain and Italy. Because each country was represented by a single institution with a distinct disciplinary composition, this split describes differences between the participating settings rather than an effect of nationality (Section 4.5).

Within each of these three branches the tree retained a different characteristic: living arrangement among students from Chile and Mexico, year of study among Spanish students, and self-reported change in PA following the pandemic within the Italian branch. These associations provide descriptive information about the characteristics with which food-consumption profiles co-occurred in each setting. They do not establish that living arrangement, year of study, or changes in PA caused differences in dietary behaviour. In particular, the association with year of study cannot be interpreted as evidence that dietary behaviour changes progressively during university, because the cross-sectional design does not allow within-person changes to be assessed. Similarly, the association between post-pandemic PA change and dietary profile membership does not establish whether changes in PA and diet occurred concurrently or were related to one another.

Within the Italian branch, students reporting similar or moderately increased PA were markedly more often assigned to the Mixed Dietary profile and markedly less often to the Low Plant-Food profile than students reporting either reduced PA or a marked increase in PA. Representation of the Healthy and Prudent and Protein-Oriented profiles differed little between nodes, indicating that the split is driven by the contrast between the Mixed Dietary and Low Plant-Food profiles rather than by a general gradient of dietary quality.

The reduced-PA component of this contrast is consistent with evidence that health-related behaviours co-occur among young adults [15,40] and with work treating diet and PA as components of a single lifestyle construct in Italian university students [53]. The grouping of students reporting a marked increase in PA with those reporting a decrease is less readily interpreted. One possibility is that it reflects more rigid or compensatory diet–activity configurations, as described in students under psychological distress [16]. Alternatively, it may be an artefact of the small number of cases in that response category and of the merging of non-adjacent categories, which the algorithm permits when the predictor is treated as nominal. The two explanations cannot be distinguished with the present data.

Because PA entered the analysis only as a candidate predictor and not as a clustering variable, this result should be read as a context-specific association within the Italian branch rather than as evidence of a combined diet–PA behavioural profile. Two further constraints apply: the PA measure is retrospective and self-reported, and it refers to change whereas food consumption was assessed as current status. Given the cross-sectional design, the temporal ordering of the two sets of behaviours cannot be established.

The findings support a context-sensitive rather than country-deterministic interpretation of student food-consumption profiles. The four configurations were observed in all participating settings, but the characteristics associated with membership varied between them. Profile identification and the examination of correlates therefore provide complementary information. The former describes the configurations of food consumption observed in the sample; the latter indicates which characteristics accompanied those configurations within each participating context. Further studies using larger, more diverse, and longitudinal samples are needed to determine whether these associations are reproducible beyond the specific institutions and populations represented here.

### 4.4. Implications for University Health Promotion

The presence of four distinct food-consumption profiles in every participating setting, combined with differences in the characteristics associated with membership, suggests that university students should not be approached as a homogeneous population when dietary interventions are designed. Interventions may benefit from attending to the characteristics locally associated with dietary profiles. The variable retained in each branch differed—living arrangement in the Chilean/Mexican branch, year of study in the Spanish branch and post-pandemic change in PA in the Italian branch—and these operate at different levels: interpersonal, academic-organisational and behavioural. Only the first two are properly contextual; the third is a co-occurring health behaviour rather than a feature of the setting.

This heterogeneity is consistent with socioecological models of healthy eating, in which individual, interpersonal, organisational and environmental factors jointly shape dietary behaviour [1,9,54], and with evidence that the university setting itself constrains students’ eating patterns [10]. From this perspective, dietary health is not solely an individual responsibility but may also be addressed through the conditions in which students study, live and make food-related decisions, an approach compatible with the whole-system framework of the Okanagan Charter [55]. The association between profile membership and post-pandemic PA change within the Italian subsample also supports examining nutrition and PA jointly rather than in isolation [56]; that this association was not monotonic—students reporting a marked increase in PA were grouped with those reporting a decrease—suggests a relationship more complex than the simple co-occurrence of favourable behaviours described in the clustering literature [15].

These implications should be read as hypotheses rather than as established intervention targets. Tree-based selection is sample-dependent, and the divergence in retained correlates may reflect genuine contextual differences or differences in branch size, statistical power and the distribution of candidate predictors. Neither the present design nor the available evidence establishes that profile-oriented or context-specific interventions outperform universal approaches, or that addressing diet and PA together yields greater benefit than addressing them separately; longitudinal and intervention studies are required to test both premises. Common nutritional objectives can therefore be retained while attention is given to the characteristics associated with dietary behaviour in particular settings.

### 4.5. Strengths, Limitations and Future Research

A principal strength of this study is the combination of person-centred profiling with subsequent exploratory characterisation of profile correlates. Previous person-centred work in university populations has pursued different aims: single-country descriptive characterisation of dietary and lifestyle habits [11,57,58]; integrated activity–diet profiling within a single national sample [16,59]; profiling anchored to a metabolic outcome [60]; and clustering based on psychological and sustainability-related eating traits rather than food-group adherence [61]. The one multicountry clustering study identified in this population used health-risk behaviours rather than food-group configurations as inputs [40]. The present analysis draws on the same four-country sample as our previous study [23], which modelled risk of poor overall diet quality from the summary IASE score; here, by contrast, multidimensional configurations of food-group adherence are derived and profile membership itself becomes the object of characterisation. To our knowledge, this configurational approach has not previously been applied across national contexts in this population.

The multicountry design is another strength. The four national subsamples were of broadly similar size and were collected using common instruments and harmonised procedures. This allowed the study to examine whether comparable food-consumption configurations could be identified across different university settings and whether the characteristics associated with profile membership were similar or different across those settings. However, the design should not be interpreted as providing nationally representative estimates or as isolating national effects.

Several limitations qualify the interpretation of the findings. First, the cross-sectional design prevents causal or temporal inference. In particular, the associations observed between profile membership and living arrangement, year of study, or self-reported post-pandemic PA change cannot be interpreted as determinants, pathways, or trajectories of dietary behaviour. Longitudinal studies are needed to establish whether food-consumption profiles remain stable during university education or change with academic progression and other life transitions.

Second, recruitment was institution-based and non-probabilistic, with one participating institution per country. The academic composition of the national samples also differed substantially between institutions, with Education-related programmes accounting for 83.1% of the total sample. Consequently, institution, academic programme, and country were partially confounded. The country split identified by CHAID therefore represents the combined context of the participating institutions, their academic composition, recruitment procedures, and broader national setting rather than an independent effect of nationality. The findings should consequently not be generalised to university students nationally or to academic disciplines that were underrepresented in the sample. Future studies should incorporate multiple institutions per country and, where feasible, probability-based or stratified sampling designs with broader representation of academic disciplines.

Third, the dietary, anthropometric, and PA measures were based on self-report and may therefore be affected by recall and social-desirability bias. The IASE provides a useful food-based measure of adherence to its operationalised food-group recommendations, but it does not assess portion sizes, nutrient intake, or meal timing. In addition, the IASE was developed from Spanish dietary recommendations and was not designed specifically as a cross-national measurement instrument. Its use provided a common framework for comparative profiling, but differences in national dietary recommendations, food cultures, and customary consumption cannot be fully captured. Formal cross-cultural measurement invariance was not assessed; consequently, cross-national differences in component scores should be interpreted cautiously.

Fourth, the clustering solution itself should be regarded as exploratory. Although the four-profile solution was substantively interpretable, it was not supported by the quantitative criteria detailed in Section 3.4 (internal validity indices, silhouette coefficients, and split-half stability, including for the retained solution). The component scores were also standardised before clustering. This gives equal weight to each food-group recommendation but amplifies the contribution of the components with the least dispersion, and solutions derived without standardisation would not necessarily coincide with those reported here. The four profiles should therefore be understood as relative configurations describing this particular sample rather than as sharply bounded or empirically established categories, and their replication in independent samples is a precondition for any further use.

The CHAID analysis presents a related limitation: it was designed for exploratory segmentation rather than individual-level prediction, and its cross-validated accuracy did not exceed the majority-class baseline (Section 3.5). The tree therefore identifies characteristics statistically associated with profile membership in this sample, and terms such as pathways or trajectories should be avoided when describing these results.

The study also has limitations regarding the interpretation of sustainability. Although several food groups represented in the IASE overlap with those included in plant-based and planetary-health dietary frameworks, environmental impacts were not directly measured. The sustainability-related interpretation is therefore conceptual and hypothesis-generating rather than an assessment of the environmental footprint of the identified profiles. Future studies should combine food-consumption profiling with explicit indicators of greenhouse-gas emissions, land and water use, or other validated measures of dietary environmental impact.

Future research should prioritise replication across independent samples, multiple institutions within each country, and more diverse academic populations. Longitudinal designs could determine whether the identified profiles are stable or change during university education, while larger samples would permit more robust examination of sex-specific and other subgroup differences. Extending the person-centred framework to include sleep, food insecurity, psychological well-being, nutritional literacy, and objectively measured PA could also clarify whether food-consumption profiles form part of broader lifestyle configurations. Finally, intervention studies should examine whether students with different food-consumption profiles respond differently to nutrition and health-promotion strategies, preferably within Health Promoting University frameworks and using implementation-oriented designs.

## 5. Conclusions

This multicountry study used a person-centred approach based exclusively on the nine IASE food-group component scores to describe food-consumption profiles among university students. Four profiles were retained, and all four were observed in each of the participating countries, indicating that comparable configurations of food-group adherence could be identified across the four university settings. This cross-setting presence should not be read as evidence that the profiles themselves are statistically reproducible: the clustering solution was exploratory, the separation between profiles was limited, and the profile boundaries were not consistently recovered in random subsamples of the present data. The characteristics associated with profile membership also differed across the national branches of the decision tree: country was the primary segmentation variable, followed by living arrangement in the Chile/Mexico branch, year of study in Spain, and self-reported post-pandemic PA change in Italy.

The main contribution of the study is methodological and cautionary rather than substantive: it shows that a superficially interpretable four-profile solution can be derived from food-group adherence scores in a multicountry university sample, but that this structure lacks internal validity and split-half replicability, and that Profile 4 does not survive comparison against a more parsimonious three-cluster solution. Among the characteristics examined, country and academic programme—themselves confounded with participating institution—showed the largest, although still modest, association with profile membership (Cramer’s V = 0.165–0.179); no other characteristic approached this magnitude. Replication of the clustering structure in probabilistic, multi-institution samples is accordingly a precondition for treating these profiles as an established dietary typology. The clustering analysis describes the dietary configurations present in the sample, whereas the subsequent Exhaustive CHAID analysis shows that their associated characteristics were not uniform across the participating settings. These findings are descriptive and exploratory and should not be interpreted as evidence of causal determinants, temporal pathways, individual-level predictive performance, or an established dietary typology. The identified profiles also differed in their relative adherence to plant-based and animal-source food groups; however, environmental impacts and dietary sustainability were not directly assessed.

Replication is therefore a precondition rather than a desirable extension. Further research should attempt to recover these profiles in independent samples, across multiple institutions and more diverse university populations, and should use longitudinal designs to determine whether such configurations are temporally stable and whether their associated characteristics are reproducible. Studies integrating dietary profiling with PA and other health behaviours, as well as direct measures of dietary environmental impact, could then clarify the relevance of these configurations for comprehensive university health-promotion strategies.

## Figures and Tables

**Table 1:** Overall characteristics of the study population (*N* = 686).

Variable	Overall Sample
Age (years), median (IQR)	21.3 (19.49–23.31)
Sex, *n* (%)	
Female	417 (60.8)
Male	269 (39.2)
Country, *n* (%)	
Chile	161 (23.5)
Mexico	162 (23.6)
Spain	180 (26.2)
Italy	183 (26.7)
Study programme, *n* (%)	
Education-related programmes	570 (83.1)
Psychology	67 (9.8)
Other programmes	49 (7.1)
Year of study, *n* (%)	
First	245 (35.7)
Second	94 (13.7)
Third	115 (16.8)
Fourth	105 (15.3)
Other	127 (18.5)
Perceived socioeconomic status, *n* (%)	
Low	55 (8.0)
Medium-low	99 (14.4)
Medium	337 (49.1)
Medium-high	100 (14.6)
High	34 (5.0)
Don’t know/No response	61 (8.9)
Living arrangement, *n* (%)	
With close family members	575 (83.8)
With other people	111 (16.2)
Employment status, *n* (%)	
Employed	263 (38.3)
Unemployed	423 (61.7)
Financial situation, *n* (%)	
Fully dependent	406 (59.2)
Partially independent (<50% of expenses)	153 (22.3)
Partially independent (>50% of expenses)	81 (11.8)
Fully independent	46 (6.7)
BMI (kg/m^2^), median (IQR)	23.0 (20.80–25.50) †
BMI category, *n* (%)	
Underweight	48 (7.0)
Normal weight	429 (62.6)
Overweight	171 (25.0)
Obesity	37 (5.4)
Currently following a medically prescribed diet, *n* (%)	
Yes	51 (7.4)
No	635 (92.6)
Current medical or psychological treatment, *n* (%)	
Medical treatment	58 (8.5)
Psychological treatment	72 (10.5)
No treatment	556 (81.0)
History of COVID-19 infection, *n* (%)	
No known infection	345 (50.3)
Self-diagnosed (self-test)	130 (19.0)
Diagnosed by a healthcare professional	211 (30.8)
Required to restrict PA during lockdown, *n* (%)	
No	273 (39.8)
Yes, due to COVID-19 physical consequences	110 (16.0)
Yes, due to other non-COVID-19 causes	211 (30.8)
Yes, due to lockdown or space restrictions	92 (13.4)
Self-reported change in PA after the pandemic, *n* (%)	
Much more physically active	141 (20.6)
More physically active	149 (21.7)
Similar physical activity	208 (30.3)
Less physically active	140 (20.4)
Much less physically active	48 (7.0)
IPAQ-SF PA classification, *n* (%)	
Low	113 (16.5)
Moderate	166 (24.2)
High	407 (59.3)

Abbreviations: BMI, body mass index; IQR, interquartile range; PA, physical activity; IPAQ-SF, International Physical Activity Questionnaire–Short Form. † Body mass index was computed from self-reported height and weight; weight status was classified according to WHO cut-offs. One participant with an invalid anthropometric record was excluded from the BMI variables, which are therefore based on 685 participants. Note: PA categories were determined using the standard IPAQ-SF classification algorithm [26,27]. Low: did not meet the criteria for moderate or high PA. Moderate: ≥3 days of vigorous PA for ≥20 min/day, or ≥5 days of moderate PA and/or walking for ≥30 min/day, or ≥5 days of any combination of PA accumulating ≥600 MET-min/week. High: ≥3 days of vigorous PA accumulating ≥1500 MET-min/week, or ≥7 days of any combination of PA accumulating ≥3000 MET-min/week.

**Table 2:** Healthy Eating Index adapted for the Spanish population component scores across the identified food-consumption profiles.

IASE Component Score (0–10 Points)	Profile 1 (*n* = 170)	Profile 2 (*n* = 89)	Profile 3 (*n* = 251)	Profile 4 (*n* = 176)
Cereals and derivatives	10.0 (7.5–10.0)	10.0 (7.5–10.0)	10.0 (10.0–10.0)	7.5 (5.0–10.0)
Vegetables and greens	10.0 (7.5–10.0)	7.5 (5.0–10.0)	10.0 (7.5–10.0)	7.5 (5.0–7.5)
Fruit	10.0 (7.5–10.0)	5.0 (5.0–7.5)	7.5 (7.5–10.0)	5.0 (5.0–7.5)
Dairy products	7.5 (5.0–10.0)	7.5 (5.0–10.0)	10.0 (7.5–10.0)	7.5 (5.0–10.0)
Meat, fish and eggs	2.5 (2.5–7.5)	7.5 (2.5–7.5)	7.5 (2.5–7.5)	7.5 (7.5–10.0)
Legumes	10.0 (7.5–10.0)	0.0 (0.0–0.0)	10.0 (7.5–10.0)	10.0 (10.0–10.0)
Processed meats and cold cuts †	5.0 (5.0–10.0)	5.0 (2.5–5.0)	5.0 (2.5–5.0)	5.0 (2.5–5.0)
Sweets †	5.0 (5.0–10.0)	2.5 (0.0–5.0)	2.5 (2.5–5.0)	5.0 (2.5–5.0)
Sugar-sweetened beverages †	10.0 (10.0–10.0)	5.0 (2.5–10.0)	5.0 (2.5–5.0)	5.0 (2.5–5.0)

Values are presented as median (interquartile range). Abbreviations: IASE, Healthy Eating Index adapted for the Spanish population. Notes: The four profiles were identified using hierarchical cluster analysis (Ward’s method) followed by k-means refinement based on the nine IASE component scores. Scores range from 0 to 10, with higher values indicating greater adherence to the corresponding IASE recommendation. † For processed meats and cold cuts, sweets, and sugar-sweetened beverages, higher scores indicate lower consumption and therefore greater adherence to the recommendation. Because the component scores were used to derive the clusters, Table 2 is intended to describe the resulting configurations rather than to provide independent statistical comparisons between profiles.

**Table 3:** Raw consumption frequency of plant-based and animal-source food groups across food-consumption profiles.

Food-Group Composite	Healthy and Prudent (Profile 1, *n* = 170) Mean (SD)	Low Plant-Food (Profile 2, *n* = 89) Mean (SD)	Mixed Dietary (Profile 3, *n* = 251) Mean (SD)	Protein-Oriented (Profile 4, *n* = 176) Mean (SD)	Effect Size
Plant-based frequency ^a^	3.21 (0.42)	2.24 (0.86)	3.23 (0.38)	2.30 (0.43)	ε^2^ = 0.476 ***
Animal-source frequency ^b^	2.46 (0.71)	2.55 (0.79)	2.99 (0.61)	2.59 (0.71)	ε^2^ = 0.099 ***

Notes. Profile labels are descriptive labels assigned post hoc from the relative configuration of the nine IASE component scores. “Protein-Oriented” refers primarily to the relative prominence of the meat, fish and eggs component and does not indicate absolute protein intake or overall animal-source food consumption. Frequency scale: 0 = never or almost never, 1 = once a week, 2 = once–twice a week, 3 = three or more times per week, and 4 = daily. ^a^ Plant-based composite: mean raw consumption frequency of cereals, vegetables, fruit, and legumes. ^b^ Animal-source composite: mean raw consumption frequency of meat/fish/eggs, processed meats and cold cuts, and dairy products. Differences across profiles were assessed using the Kruskal–Wallis test; ε^2^, epsilon squared; *** *p* < 0.001. The composites are based on the same underlying food-group variables used to derive the clusters and therefore constitute a descriptive characterisation rather than an independent validation of profile membership.

**Table 4:** Sociodemographic and lifestyle characteristics showing significant differences across the identified food-consumption profiles.

Variable	Healthy and Prudent (Profile 1, *n* = 170) *n* (%)	Low Plant-Food (Profile 2, *n* = 89) *n* (%)	Mixed Dietary (Profile 3, *n* = 251) *n* (%)	Protein-Oriented (Profile 4, *n* = 176) *n* (%)	Test, *p* and Effect Size
Age (years), median (IQR)					H(3) = 29.83, *p* < 0.001, ε^2^ = 0.039
	22.09 (20.02–23.96)	22.39 (20.34–23.84)	21.02 (19.55–23.09)	20.25 (19.01–22.63)	
Country					χ^2^(9) = 55.81, *p* < 0.001, *V* = 0.165
Chile	28 (16.5)	19 (21.3)	73 (29.1)	41 (23.3)	
Mexico	26 (15.3)	17 (19.1)	66 (26.3)	53 (30.1)	
Spain	53 (31.2)	13 (14.6)	58 (23.1)	56 (31.8)	
Italy	63 (37.1)	40 (44.9)	54 (21.5)	26 (14.8)	
Academic programme					χ^2^(21) = 66.09, *p* < 0.001, *V* = 0.179
Clinical Psychology	22 (12.9)	16 (18.0)	19 (7.6)	10 (5.7)	
Early Childhood Education	4 (2.4)	0 (0.0)	5 (2.0)	6 (3.4)	
Educational Psychology	34 (20.0)	16 (18.0)	27 (10.8)	11 (6.3)	
Educational Therapy	9 (5.3)	4 (4.5)	19 (7.6)	15 (8.5)	
Others (Social Communication, Social Education, Philosophy)	12 (7.1)	11 (12.4)	17 (6.8)	9 (5.1)	
Pedagogy	18 (10.6)	12 (13.5)	51 (20.3)	48 (27.3)	
Physical Education Pedagogy	28 (16.5)	19 (21.3)	73 (29.1)	41 (23.3)	
Primary Education	43 (25.3)	11 (12.4)	40 (15.9)	36 (20.5)	
Year of study					χ^2^(12) = 45.91, *p* < 0.001, *V* = 0.149
Year 1	42 (24.7)	27 (30.3)	91 (36.3)	85 (48.3)	
Year 2	35 (20.6)	8 (9.0)	33 (13.1)	18 (10.2)	
Year 3	21 (12.4)	13 (14.6)	50 (19.9)	31 (17.6)	
Year 4	40 (23.5)	18 (20.2)	32 (12.7)	15 (8.5)	
Year 5	32 (18.8)	23 (25.8)	45 (17.9)	27 (15.3)	
IPAQ-SF PA classification					χ^2^(6) = 28.04, *p* < 0.001, *V* = 0.143
Low	17 (10.0)	30 (33.7)	41 (16.3)	25 (14.2)	
Moderate	47 (27.6)	22 (24.7)	59 (23.5)	38 (21.6)	
High	106 (62.4)	37 (41.6)	151 (60.2)	113 (64.2)	
Currently on a medically prescribed diet					χ^2^(3) = 18.62, *p* < 0.001, *V* = 0.165
No	145 (85.3)	82 (92.1)	240 (95.6)	168 (95.5)	
Yes	25 (14.7)	7 (7.9)	11 (4.4)	8 (4.5)	
Change in PA level after the COVID-19 pandemic					χ^2^(12) = 26.89, *p* = 0.008, *V* = 0.114
Much more active	36 (21.2)	19 (21.3)	48 (19.1)	38 (21.6)	
More active	35 (20.6)	12 (13.5)	67 (26.7)	35 (19.9)	
No change	63 (37.1)	21 (23.6)	77 (30.7)	47 (26.7)	
Less active	24 (14.1)	25 (28.1)	49 (19.5)	42 (23.9)	
Much less active	12 (7.1)	12 (13.5)	10 (4.0)	14 (8.0)	

Abbreviations: IQR, interquartile range; V, Cramer’s V; ε^2^, epsilon squared; PA, physical activity; IPAQ-SF, International Physical Activity Questionnaire–Short Form. Notes: Values are presented as median (interquartile range) for age and as n (%) for categorical characteristics. Percentages are within-profile (column) percentages: they express the proportion of the members of each profile falling into a given category and sum to 100% down each profile column. Percentages describing the distribution of profiles within countries (within-row percentages) are reported in the text where relevant. Age was compared across profiles using the Kruskal–Wallis test (H), followed by Dunn–Bonferroni pairwise comparisons; categorical characteristics were compared using Pearson’s chi-square test (χ^2^), with adjusted standardised residuals used to identify the cells contributing to each association. Effect sizes are reported as epsilon squared (ε^2^) for the Kruskal–Wallis test and Cramer’s V for the chi-square tests, the latter interpreted according to the effective degrees of freedom of each table, defined as min(rows − 1, columns − 1). The *p*-values shown are unadjusted; values corrected for multiple comparisons across the full family of 17 profile-characterisation tests using the Benjamini–Hochberg false discovery rate procedure are reported in Supplementary Table S3. The table presents only the characteristics that remained significantly associated with profile membership after that correction; the remaining ten are reported in Supplementary Table S3.

## Data Availability

The data supporting the findings of this study are available from the corresponding author upon reasonable request.

## Supplementary Materials

The following supporting information can be downloaded at https://www.mdpi.com/article/10.3390/nu18172869/s1. Table S1: Cluster-quality and split-half stability indices for two- to five-cluster solutions; Table S2: Correspondence between the selected four-cluster solution and the alternative three-cluster solution; Table S3: Associations between participant characteristics and food-consumption profile membership, and Profile 4–specific patterns.

## Abbreviations

The following abbreviations are used in this manuscript:

ARI	Adjusted Rand Index
BMI	Body Mass Index
CART	Classification and Regression Tree
CHAID	Chi-square Automatic Interaction Detection
COVID-19	Coronavirus Disease 2019
FDR	False Discovery Rate
HEI	Healthy Eating Index
IASE	Índice de Alimentación Saludable para la población Española
IPAQ-SF	International Physical Activity Questionnaire—Short Form
IQR	Interquartile Range
MET	Metabolic Equivalent of Task
PA	Physical Activity
SD	Standard Deviation
SE	Standard Error
STROBE	Strengthening the Reporting of Observational Studies in Epidemiology
WHO	World Health Organization
