# Supplementary material for: Food-Consumption Profiles and Context-Specific Associations Among University Students: An Exploratory Multicountry Person-Centred Clustering and CHAID Analysis
Source: Nutrients. 2026 Sep 2;18(17):2869. doi: 10.3390/nu18172869 (PMC13567024; doi:10.3390/nu18172869)
Supplement: Supplementary file 1 [file nutrients-18-02869-s001.zip › Supplementary Table S1.pdf]

Table S1. Cluster-quality and split-half stability indices for two- to five-cluster solutions.

Number of clusters (k)	Calinski–Harabasz index	Davies–Bouldin index	Silhouette coefficient	Adjusted Rand Index (M, SD)	Cramer's V (stability)	Misclassification rate
2	90.1	2.618	0.113	0.10 (0.21)	0.22	37.4%
3	83.7	2.271	0.126	0.28 (0.18)	0.53	37.0%
4 (selected solution)	80.9	2.072	0.113	0.30 (0.10)	0.58	40.2%
5	74.9	1.916	0.118	0.35 (0.10)	0.59	40.6%

Note: The nine IASE component scores were standardised (z-scores) before clustering; all indices reported in this table were computed on the standardised scores. Profiles were identified using Ward's hierarchical clustering with squared Euclidean distance followed by k-means refinement, with the hierarchical cluster centroids used as initial seeds. Higher Calinski–Harabasz and silhouette values indicate better cluster separation, whereas lower Davies–Bouldin values indicate better cluster compactness and separation. Split-half stability was assessed across 100 random 50/50 sample partitions. Within each partition, clustering was performed independently in each half, after which observations in each half were assigned to the cluster centroids derived from the opposite half using the nearest-centroid rule. Agreement between the native clustering solution and the corresponding cross-assigned classification was quantified using the Adjusted Rand Index (ARI), Cramer's V, and the misclassification rate, after optimal alignment of cluster labels using the Hungarian algorithm. Reported split-half stability indices represent the mean across the two directional cross-assignment procedures and the 100 random partitions.
