# Supplementary material for: Food-Consumption Profiles and Context-Specific Associations Among University Students: An Exploratory Multicountry Person-Centred Clustering and CHAID Analysis
Source: Nutrients. 2026 Sep 2;18(17):2869. doi: 10.3390/nu18172869 (PMC13567024; doi:10.3390/nu18172869)
Supplement: Supplementary file 1 [file nutrients-18-02869-s001.zip › Supplementary Table S2.pdf]

Table S2. Correspondence between the selected four-cluster solution and the alternative three-cluster solution.

Three-cluster solution	From Profile 1 (n = 170)	From Profile 2 (n = 89)	From Profile 3 (n = 251)	From Profile 4 (n = 176)	Total (n)
Cluster A	0	89	13	1	103
Cluster B	169	0	44	28	241
Cluster C	1	0	194	147	342

Note: The three-cluster solution was derived from the same standardised IASE component scores and the same clustering procedure as the retained four-cluster solution. Cell values indicate the number of participants belonging to each profile in the selected four-cluster solution who were classified into each cluster of the alternative three-cluster solution. Cluster A corresponds predominantly to Profile 2 and is characterised by very low adherence to the legumes component (median 0.0). Cluster B corresponds predominantly to Profile 1 and is characterised by high adherence to vegetables and fruit together with the most favourable scores for the components recommended to be limited, and by the lowest adherence score for meat, fish and eggs. Cluster C corresponds predominantly to Profile 3 and is characterised by high adherence to cereals, legumes and the meat, fish and eggs component, together with comparatively lower adherence to processed meats and cold cuts, sweets, and sugar-sweetened beverages. Profile 4 was not preserved as a distinct configuration in the three-cluster solution: 147 of its members (83.5%) were assigned to Cluster C and 28 (15.9%) to Cluster B, with a single case assigned to Cluster A. Cluster A, Cluster B, and Cluster C refer exclusively to the alternative three-cluster solution and are not equivalent to the four profiles retained in the primary analysis.
