# Supplementary material for: Food-Consumption Profiles and Context-Specific Associations Among University Students: An Exploratory Multicountry Person-Centred Clustering and CHAID Analysis
Source: Nutrients. 2026 Sep 2;18(17):2869. doi: 10.3390/nu18172869 (PMC13567024; doi:10.3390/nu18172869)
Supplement: Supplementary file 1 [file nutrients-18-02869-s001.zip › Supplementary Table S3.pdf]

Table S3. Associations between participant characteristics and food-consumption profile membership, and Profile 4–specific patterns.

Variable	Test	Statistic	df	p (raw)	p (FDR-adjusted)	Effect size	Profile 4–specific pattern
Country	χ^2	55.81	9	<0.001	<0.001	V = 0.165	Overrepresented among Mexican students (adj. residual = 2.35); underrepresented among Italian students (−4.14).
Academic programme	χ^2	66.09	21	<0.001	<0.001	V = 0.179	Overrepresented in Pedagogy (3.33); underrepresented in Clinical Psychology (−2.12) and Educational Psychology (−3.03).
Age	H	29.83	3	<0.001	<0.001	ε^2 = 0.039	Profile 4 was younger than Profiles 1, 2 and 3 (Dunn–Bonferroni adjusted $p < 0.001$, $p < 0.001$ and $p = 0.039$, respectively); no other pairwise comparison was significant.
Year of study	χ^2	45.91	12	<0.001	<0.001	V = 0.149	Overrepresented among first-year students (4.04); underrepresented among fourth-year students (−2.90).
Habitual physical activity level	χ^2	28.04	6	<0.001	<0.001	V = 0.143	No significant adjusted residual involved Profile 4; the global association was attributable to differences among Profiles 1 and 2.
Prescribed diet	χ^2	18.62	3	<0.001	<0.001	V = 0.165	No significant adjusted residual involved Profile 4; the global association was attributable to differences among Profiles 1 and 3.
Physical activity change post-pandemic	χ^2	26.89	12	0.008	0.019	V = 0.114	No significant adjusted residual involved Profile 4; the global association was attributable to differences among Profiles 1, 2 and 3.
Living arrangement	χ^2	7.11	3	0.068	0.145	V = 0.102	—
Financial situation	χ^2	15.39	9	0.081	0.153	V = 0.086	—
Sex	χ^2	6.07	3	0.108	0.184	V = 0.094	—
Perceived socioeconomic status	χ^2	20.65	15	0.149	0.217	V = 0.100	—
Current medical or psychological treatment	χ^2	9.32	6	0.156	0.217	V = 0.082	—

Variable	Test	Statistic	df	p (raw)	p (FDR-adjusted)	Effect size	Profile 4–specific pattern
Pandemic-related PA restriction	χ^2	12.92	9	0.166	0.217	V = 0.079	—
COVID-19 history	χ^2	8.54	6	0.201	0.244	V = 0.079	—
Weight status (BMI category)	χ^2	10.30	9	0.327	0.371	V = 0.071	—
Employment status	χ^2	1.28	3	0.735	0.781	V = 0.043	—
BMI	H	0.76	3	0.860	0.860	$\varepsilon^2 < 0.001$	—

Note: Variables are ordered by Benjamini–Hochberg-adjusted p-value. Associations between categorical characteristics and food-consumption profile membership were assessed using Pearson's chi-square test (χ^2); age and body mass index (BMI) were compared across profiles using the Kruskal–Wallis test (H). The Benjamini–Hochberg false discovery rate procedure was applied jointly to the full family of 17 profile-characterisation tests (15 chi-square tests and two Kruskal–Wallis tests) at a false discovery rate of 0.05; seven characteristics remained significant after correction. Effect sizes are reported as Cramer's V for categorical characteristics, with its magnitude interpreted according to conventional benchmarks considering the effective degrees of freedom of each contingency table, defined as min (rows − 1, columns − 1), and as epsilon squared (ε^2) for the Kruskal–Wallis tests. Adjusted standardised residuals with $|z| \geq 1.96$ were considered indicative of a deviation from the expected cell frequency under independence; these residuals were inspected only for characteristics that remained significant after correction and were not further adjusted for multiplicity, and are therefore exploratory. The "Profile 4–specific pattern" column reports only significant residuals involving Profile 4 (or, for age, the significant Dunn–Bonferroni pairwise comparisons involving Profile 4); an em dash indicates that the characteristic was not significantly associated with profile membership after correction and was therefore not examined at cell level. Where no residual involving Profile 4 reached the $|z| \geq 1.96$ threshold, the significant global association was driven by differences among the other profiles and therefore did not provide evidence that Profile 4 was specifically differentiated by that characteristic. BMI and weight status were computed for the 685 participants with a valid anthropometric record. All reported associations were small to moderate in magnitude.
